# Debridement and primary closure of a mesenteric duodenal perforation in a dog

**DOI:** 10.1002/vms3.1157

**Published:** 2023-05-18

**Authors:** Laura Edwards, Beatriz Belda, Marije Risselada

**Affiliations:** ^1^ Department of Veterinary Clinical Sciences, College of Veterinary Medicine Purdue University West Lafayette Indiana

**Keywords:** duodenal perforation, linear foreign body, primary closure

## Abstract

A 7‐year‐old male mixed intact breed dog was presented with a 6‐day history of lethargy and anorexia. A linear foreign body was diagnosed and an exploratory laparotomy performed. The foreign body was pushed orad and removed via gastrotomy. Two mesenteric duodenal perforations were found: one at the level of the common bile duct and a second at the duodenal flexure. Both lesions were debrided and primarily closed in a simple interrupted appositional pattern. A gastrostomy tube and closed suction drain were placed routinely. The dog recovered without complications and ate voluntarily the first day postoperatively. The drain and gastrostomy tube were removed without incident at 4 and 15 days, respectively. Five months postoperatively the dog was reported to be clinically normal. Debridement and primary closure may represent an alternative to more extensive surgery with rerouting for duodenal perforations in select cases.

## Graphical abstract

Mesenteric duodenal perforations in a dog was debrided and closed primarily, avoiding the need for more invasive rerouting procedures for the perforation at the level of the common bile duct. Arrow heads: perforations (white: at the level of the common bile duct, yellow: duodenal flexure); P: pylorus; *: pancreatic body. The dog went on to make a full recovery without surgical site complications.

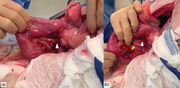

## 1 INTRODUCTION

The orad section of the duodenum has specific anatomical surgical considerations, due to the common bile duct and pancreatic connecting to the intestinal lumen as well as the proximity of the pancreatic ducts. Removal of a lesion in the proximal duodenum close to the pylorus would necessitate a pylorectomy, proximal duodenectomy and biliary rerouting via a cholecystoenterostomy (Walter et al., 1985). These procedures have been described as having a high morbidity and mortality rate in the veterinary literature. This was consolidated by the author's personal experience with a negative outcome of a Billroth II. Cholecystoenterostomy alone has been associated with hepatic abscesses, pancreatitis, vomiting as well as acquired portosystemic shunts in dogs (Papazoglou et al., 2008). Surgical diseases of this segment might therefore lead to a different decision making process than those further aborad due to concerns regarding patient morbidity and/or mortality postoperatively. Two perforations due to a linear foreign body (LFB) obstruction were identified in the orad section of the duodenum of a dog and treated with debridement and primary repair. To the authors' knowledge, there are no other reports of successful debridement and primary repair of multiple mesenteric perforations of the orad duodenum.

### 1.1 Case history

A 27 kg, 7‐year‐old mixed breed male intact canine was evaluated for lethargy, anorexia, weight loss and absence of defecation for 6 days.

On presentation, the dog had increased skin turgor, pale mucous membranes with increased capillary refill time (3 s) and mild grit in faecal matter on rectal exam. Clinical laboratory tests revealed severe hypokalaemia (2.6 mmol/L, reference range 3.5–5.0 mmol/L), mild hypocalcaemia (8.4 mg/dL, reference range 9.7–12.3 mg/dL), hypophosphataemia (1.9 mg/dL, reference range 2.2–7.9), hyponatraemia (128 mmol/L, reference range 138–148 mmol/L) and hypochloraemia (87 mmol/L, reference range 105–117 mmol/L), with a mild inflammatory leukogram (17.3 K/μL, reference range 6.0‐17.0 K/μL), band neutrophilia (15.6 K/μL, reference range 3.0‐12.0 K/μL) and lymphocytopaenia (0.5K/μL, reference range 1.0–5.0 K/μL) (Cornell 2016; Lin et al., 2017). Abdominal radiographs were suspicious for increased gastric fluid and mild focal intestinal dilation with probable fluid contents, leading to suspicion of proximal small intestinal obstruction. Focal gastrointestinal ultrasound confirmed the presence of a gastric and duodenal LFB, and surgical abdominal exploratory surgery was recommended.

Acepromazine 0.01 mg/kg intravenously (IV) (10 mg/mL, Acepromazine Maletate Injection, Vet One) and hydromorphone 0.11 mg/kg IV (2 mg/mL, Hydromorphone HCl Injection, West‐Ward) were used for premedication. General anaesthesia was induced with propofol to effect, 2.9 mg/kg IV (10 mg/mL, Propoflo Injectable, Zoetis) and oral endotracheal intubation performed. Anaesthesia was maintained with isoflurane (Fluriso, Vet One) delivered in oxygen. Perioperative analgesia consisted of a fentanyl bolus (50 mg/mL, Fentanyl citrate injection, USP; West‐Ward) at 5 μg/kg IV followed by a constant rate infusion of 5 mg/kg/h IV. Dexmedetomidine (0.5 mg/mL Dexdomitor, Zoetis) was administered at 5 μg/kg intramuscularly (IM). Intraoperative hypotension was noted, with an invasive mean arterial pressure (MAP) of 45 mmHg recorded, and colloid therapy was started with a 3 mL/kg IV bolus followed by 3 mL/kg/h IV continuous rate infusion (CRI) of 6% hydroxyethyl starch 130/4 in 0.9% sodium chloride (Vetstarch, Zoetis). In addition, dobutamine (12.5 mg/mL Dobutamine Injectable, Hospira) was administered between 10 and 20 μg/kg/min CRI to effect to maintain a MAP of >60 mmHg.

**FIGURE 1:**
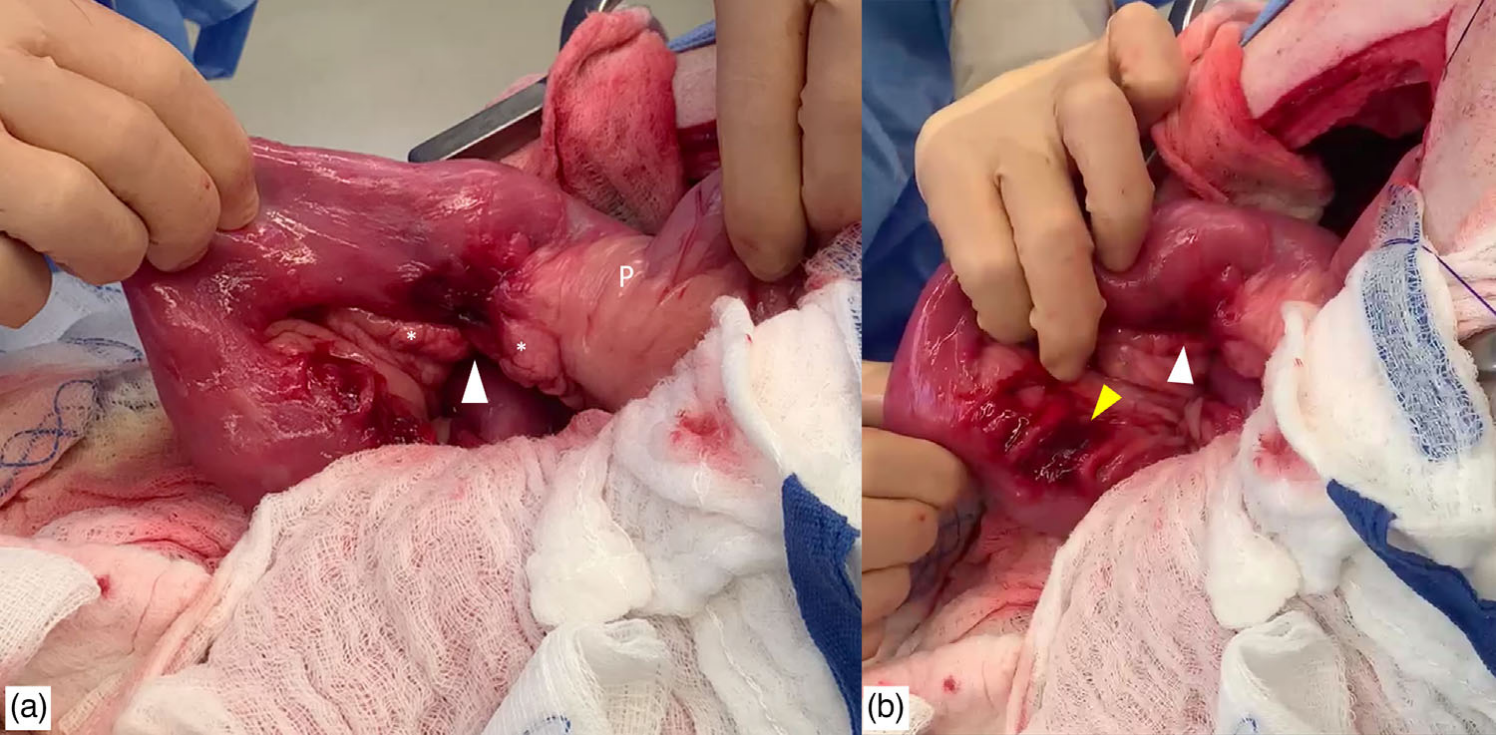
Intraoperative images showing the two lesions. (a) The surgeon is retracting the proximal duodenum, showing the more orad perforation, indicated by a white arrowhead. The pylorus (P) and body of the pancreas (*) are indicated. (b) The surgeon is retracting the duodenum towards the duodenocolic ligament, the aborad perforation is indicated by a yellow arrowhead.

The patient was positioned in dorsal recumbency and the abdomen clipped and prepared for a ventral midline abdominal approach. A complete abdominal exploratory celiotomy was performed. No free fluid was detected in the abdomen. A LFB was identified anchored at the pylorus, extending to just aborad to the duodenal flexure leading to plication and local discoloration of the intestinal wall. The LFB was removed via routine gastrotomy. Two mesenteric duodenal perforations were noted after reducing the plication: one aborad to the pylorus at the level of the minor duodenal papilla and one orad to the flexure (Figure 1). The client was contacted and declined pylorectomy with biliary diversion but approved a more conservative surgical approach. The pancreas was bluntly dissected free from the duodenum along the proximal perforation. The edges of both perforations were sharply debrided using metzenbaum scissors until the edges appeared subjectively healthy (active bleeding, no thickening on palpation), and closed primarily in a simple interrupted appositional pattern using 3‐0 polydioxanone (PDS II, Johnson & Johnson). After leak‐testing both closures, the abdomen was copiously lavaged with warm sterile saline and a left sided gastrostomy tube (24 Fr Pezzer, Bard, Inc) was placed with simple interrupted pexy sutures to the body wall using 2‐0 polydioxanone. A 7 mm Jackson Pratt (JP) drain with a 400 mL reservoir (Medline Industries) was placed prior to routine abdominal closure. The linea alba was closed using a simple continuous pattern in 0 polydioxanone, the subcutaneous tissue was apposed with a simple continuous pattern using 3‐0 poliglecaprone 25 (Monocryl, Johnson & Johnson) and the skin was closed with a continuous intradermal pattern in 3‐0 poliglecaprone 25. Individual fingertrap sutures using 2‐0 nylon (Ethilon, Johnson & Johnson) were used to secure each tube externally to the skin.

Ampicillin/sulbactam 30 mg/kg IV (Unasyn, Piramal Healthcare) was started during surgery and continued three times a day for 3 days, and 10 mg/kg IV enrofloxacin (Baytril, Bayer) was administered for 3 days while in hospital. The dog was maintained on intravenous crystalloid fluids (60 mL/kg/day, Plasmalyte A Injectable, Baxter) for 5 days. During the first evening, a low blood glucose (BG) was detected (45 mg/dL automated, and 41 mg/dL manual) and a 0.5 mg/kg dextrose bolus (Dextrose 50%, Vet One in a 1:1 dilution) was given followed by 2.5% dextrose added to the crystalloids. Repeat BG values were 74 mg/dL (after 2 h), 101 mg/dL (4 h), 137 mg/dL (8 h), 95 mg/dL (11 h) and 92 mg/dL (14 h) and remained within normal limits thereafter. The dextrose addition was discontinued after 36 h. Fentanyl was continued postoperatively at 3 μg/kg/h IV for 3 days, then tapered to 2 μg/kg/h IV for 1 day and discontinued the following day (Fentanyl, Hospira). Other medications were maropitant (1 mg/kg IV once a day for 4 days; Cerenia, Zoetis), metoclopramide (2 mg/kg/day IV CRI for 3 days; Metoclopramide, Hospira), ondansetron (0.5 mg/kg IV three times a day for 2 days; Ondansetron, Fosun) and pantoprazole (1 mg/kg IV twice a day for 3 days; Pantoprazole, Auromedics).

Postoperative abdominal fluid was monitored for volume and daily cytological appearance using the JP drain. No intracellular bacteria were seen at any time point. The fluid amount was 31, 28 and 12 mL/kg/24 h during the first, second and third postoperative days; and on day 4, the JP drain was removed. The dog was discharged from the hospital at day 5 with ampicillinsulbactam 15.6 mg/kg mg/kg per os (PO) twice daily for 154 days (375 mg Clavamox Oral Chewable, Zoetis), enrofloxacin 5.6 mg/kg PO once daily for 14 days (136 mg Baytril Oral Chewable, Bayer), gabapentin 12.5 mg/kg PO every 8–12 h for 10 days (300 mg tablet, American Health Packaging), cisapride 0.4 mg/kg PO every 8 h for 4 days (10 mg oral capsule, compounded), omeprazole 0.83 mg/kg PO every 8 h for 5 days (20 mg capsule, Dr Reddy) and trazodone 5.2 mg/kg PO every 8 h as needed (1 ¼ of 100 mg tablet, Teva). The dog returned for a recheck 15 days postoperatively. The physical exam was unremarkable, the incision site had healed (no signs of inflammation were noted) and no discomfort was noted on abdominal palpation. Body weight was 24 kg (12% body weight loss from initial presentation). The owner reported normal appetite and bowel movements and no episodes of vomiting or regurgitation, and the gastrostomy tube was removed at this time. A telephone follow‐up was performed 5 months postoperatively, and the client reported the dog to be clinically normal.

## 2 DISCUSSION

Lesions in the proximal duodenum might require pylorectomy and biliary rerouting if the major and minor duodenal papilla and common bile duct are involved. This was considered as the initial option, but not performed due to potential perioperative complications and concerns for long‐term impact on the dog's health. Perioperative complications of pylorectomy can include septic peritonitis, anorexia, vomiting coagulated blood or bilious vomiting, reflux, acute gastric rupture and gastritis (Ahmadu‐Suka et al., 1988). Mortality rate of pylorectomy has been reported as 25% mortality within 14 days postoperatively (Eisele et al., 2010). Long‐term health issues have been described as decreased body mass from malabsorption and maldigestion, gastrointestinal ulcers, duodenal obstruction and gastritis (Ahmadu‐Suka et al., 1988) as well as afferent loop syndrome (Monnet, 2020). Of 15 dogs that underwent a cholecystoenterotomy procedure, 9 died within 20 days, and of the remaining 9 dogs, 2 were lost to follow‐up after 6 days (Papazoglou et al., 2008). A total of 11 dogs had died, for 8 of whom the cause of death was directly related to the surgery or the underlying hepatobiliary disease.

Risks of debridement and primary closure range between luminal narrowing if too aggressive and dehiscence if the debridement did not reach sufficiently healthy tissue. We opted for piecemeal debridement and removing strips of intestinal wall, until such time that the tissue had the subjective appearance of noncompromised intestinal wall during the cut, and bled afterwards. Care was taken during suturing to assess force needed to pass the needle, and whether the suture would appear to ‘pull through’ the tissue. While a ‘waist’ was appreciated post repair, this did subjectively not seem enough to cause intestinal obstruction, but might be a concern in smaller dogs and cats. A duodenal sparing surgical option was recently described using a vascularised jejunal graft to close an antimesenteric duodenal defect after wide debridement while avoiding luminal narrowing (Putterman et al., 2019). Given the mesenteric location of the perforation, a debridement wide enough to raise concern about luminal narrowing would also have necessitated biliary rerouting due to the proximity of the common bile duct. We therefore opted for primary closure as opposed to adding a graft to the surgery site, due to increased surgical time, adding a second surgery (donor) site and a longer enterotomy closure line at the location of the perforation.

Omental or serosal patching could have been added to augment repairs. Given the location of the defect, the mesenteric tissue fully covered the surgery site, and the decision was made to use the natural mesenteric blood supply instead of an omental patch. Given the small approach to the intestinal wall, we opted against serosal patching as it would have necessitated clearing a larger section in order to secure the loop of intestine orad and aborad from the tissue immediately adjacent to the repair and to avoid compression of the pancreas.

Due to its hypertonicity, 50% dextrose can cause phlebitis as well as thrombosis. If given peripherally, it should be given slowly (Hospira, 2023) and diluted (BSAVA, 2023). We provided an initial diluted bolus of 50% dextrose, followed by a continuous rate infusion. Given the lack of central line access, and the low BG in our patient, we opted to dilute the initial bolus and provide immediate administration peripherally as opposed to sedation and jugular catheter placement prior to treatment.

Part of the intraoperative instrumentation for postoperative management involved placement of a JP drain and gastrostomy tube. As these both carry a risk for postoperative complications, the benefits of placement should be weighed against the risks involved. In the authors’ experience, a JP drain would allow early detection of postoperative dehiscence by an increase in abdominal effusion, as well as finding intracellular bacteria. Given the location of the lesion and the surgical manipulation of the pancreas added to the preexisting local inflammation, the authors felt that the benefits outweighed potential complications. Benefits include measuring gastric residual volume, emptying the stomach (and thereby decreasing the risk for regurgitation and potentially aspiration) and early feeding. Complications described secondary to gastrostomy tubes are early removal of the gastrostomy tube, stoma site infection and leakage around the tube into the abdomen. However, in a recent study complications of surgically placed gastrostomy were limited to minor complications in dogs with septic peritonitis (11/43) (Elmenhorst et al., 2020) with discharge around the tube being the most common issue (10/11). This reflected the findings in an earlier study on 24 dogs with surgically placed gastrostomy tubes (Hansen et al., 2019). Eight dogs experienced tube related problems: six were minor and did not require tube removal. In two dogs, the tube had migrated into the subcutaneous space, but no intraabdominal leakage or complication was seen in either.

Careful debridement and primary closure of intestinal perforations might provide an alternative surgical option for select cases where morbidity of more complex surgeries may be prohibitive.

## AUTHOR CONTRIBUTIONS

Laura Elizabeth Edwards: writing – original draft. Beatriz Belda: conceptualisation; investigation; writing – review & editing. Marije Risselada: conceptualisation; supervision; validation; visualisation; writing – review & editing.

## CONFLICT OF INTEREST STATEMENT

The authors declare no conflict of interest.

### ETHICS STATEMENT

The authors confirm that the ethical policies of the journal, as noted on the journal's author guidelines page, have been adhered to. No ethical approval was required as this is a case report where decision making was driving by best clinical practice and with full client consent.

### PEER REVIEW

The peer review history for this article is available at https://www.webofscience.com/api/gateway/wos/peer‐review/10.1002/vms3.1157.

## Data Availability

Data sharing is not applicable to this article as no new data were created or analysed in this case report.
